# Participatory approaches for developing a practical handbook integrating health information for supporting individuals with mild cognitive impairment and their families

**DOI:** 10.1111/hex.13870

**Published:** 2023-09-19

**Authors:** Yujiro Kuroda, Aya Goto, Taiki Sugimoto, Kosuke Fujita, Kazuaki Uchida, Nanae Matsumoto, Hiroyuki Shimada, Rei Ohtsuka, Minoru Yamada, Yoshinori Fujiwara, Aya Seike, Madoka Hattori, Gabin Ito, Hidenori Arai, Takashi Sakurai

**Affiliations:** ^1^ Department of Prevention and Care Science Center for Development of Advanced Medicine for Dementia, National Center for Geriatrics and Gerontology Obu Japan; ^2^ Fukushima Medical University School of Medicine, Center for Integrated Science and Humanities Fukushima Medical University Fukushima Japan; ^3^ Department of Preventive Gerontology Center for Gerontology and Social Science, National Center for Geriatrics and Gerontology Obu Japan; ^4^ Department of Epidemiology of Aging Center for Development of Advanced Medicine for Dementia, National Center for Geriatrics and Gerontology Obu Japan; ^5^ Graduate School of Comprehensive Human Sciences, University of Tsukuba Tokyo Japan; ^6^ Research Team for Social Participation and Community Health Tokyo Metropolitan Institute of Gerontology Tokyo Japan; ^7^ Graduate School of Sport and Health Science Ritsumeikan University Kyoto Japan; ^8^ Wildlife Research Center Kyoto University Kyoto Japan; ^9^ Department of Media Creation Kyoto Seika University Kyoto Japan; ^10^ National Center for Geriatrics and Gerontology Obu Japan

**Keywords:** dementia, health information, health literacy, mild cognitive impairment, participatory approach

## Abstract

**Aim:**

This study aimed to develop a patient‐centred handbook that integrates information on lifestyle modifications and psychological support strategies for individuals with mild cognitive impairment (MCI). This article provides a comprehensive record of the development process.

**Methods:**

We adopted a participatory research model for the methodology, which comprised five phases and involved an interdisciplinary team specializing in dementia and health literacy. Data were initially collected via interviews conducted among patients with MCI (*n* = 5) and their families (*n* = 5). Given the study's preliminary nature, depth and richness of the qualitative data were the key concerns for determining the sample size, rather than broad generalizability. We ensured the inclusion of diverse experiences and perspectives by facilitating the creation of patient questions (PQs) that merged scientific evidence with patient perspectives. To enhance the handbook's accessibility and utility, we continuously evaluated the same using patient interviews, health literacy tool assessments and team discussions. This comprehensive approach harmonized scientific knowledge and patient experience, leading to the development of a personalized MCI management guide.

**Results:**

The handbook comprises nine domains, encompassing 38 selected PQs: MCI, lifestyle, lifestyle‐related diseases, exercise, nutrition, social participation, cognitive training, psychological care and family support. The health literacy handbook was evaluated based on Clear Communication Index scores. The results revealed that 73.7% of the PQs were deemed difficult prerevision, whereas only 5.3% remained challenging postrevision. The formative evaluation underscored the need for more detailed explanations prerevision, whereas postrevision comments focused primarily on editorial suggestions.

**Conclusion:**

The inclusion of patients' perspectives right from the outset ensured that the handbook met their specific needs. The final version, which reflects all stakeholders' inputs, is now slated for imminent publication.

**Patient or Public Contribution:**

Patients and the public participated extensively throughout the project, from initial interviews to material evaluation and refinement.

## 1 INTRODUCTION

Japan, characterized by one of the world's longest‐lived populaces, comprises the highest percentage of older people globally. By the mid‐21st century, it is expected to transform into a super‐aging society, a phenomenon that has never been experienced before. Given that dementia cases are projected to increase significantly over the next 30 years globally,^1^ it is an important public health concern for the aging population. The intermediate state between normal cognitive function and dementia is known as mild cognitive impairment (MCI), which can be reversed to normal cognitive function.^2^ Therefore, while stiving to delay the progression of cognitive dysfunction, targeting MCI can help prevent dementia. To date, no commonly used drugs have been effective in inhibiting MCI progression, although multifactorial interventions comprising aerobic exercise, mental activity and social participation have been shown to reduce the risk of cognitive decline.^3^ In clinical practice, most cases do not receive adequate daily lifestyle guidance to delay MCI progression.

In Japan's community‐based integrated care system, the effective utilization of limited healthcare resources requires coordination not only among hospital outpatient and inpatient units but also welfare facilities, home‐visit care services and mutual support activities in neighbourhoods.^4^ Dementia is a long‐term prognosis requiring continuous care from the preclinical stage to the end of life; therefore, there is an increasing need for health information among patients, their families and professionals across multiple medical and welfare sectors.^5^ Health literacy is the key to facilitating collaboration between patients, families and professionals. Health literacy has been defined as the ‘degree to which individuals have the capacity to obtain, process and understand basic health information and services needed to make appropriate health decisions’.^6^ It plays an important role in enhancing the utilization of health information by patients and families while also enabling healthcare professionals to better communicate health‐related information.^7^ In Europe, approximately 48% of the general population demonstrates low health literacy, while in Japan, the proportion is 85%.^8^ Although this finding is not specific to older adults, it is pertinent to ensure that individuals experiencing cognitive decline can understand health information easily. Improving health literacy is strongly associated with better health outcomes, and providing health information to older adults in a simple and informative manner is necessary to promote health and prevent functional decline.^9^ A systematic review conducted in 2019 reported that lower levels of health literacy were associated with a significantly higher risk of developing dementia subsequently.^10^ The concept of health literacy encompasses healthcare professionals' ability to make health information accessible to the public, which requires effective collaboration among healthcare professionals at both the organizational and individual levels. Therefore, health literacy should be promoted through iterative processes involving interaction between the public and healthcare professionals.

In dementia care, the role of dementia literacy—defined as knowledge and beliefs that facilitate dementia recognition, management and prevention—is paramount for effective risk communication and decision‐making.^11^ Emphasizing this need, Raymond highlights the importance of enhancing dementia literacy among caregivers to ensure they can provide the highest level of care.^11^ Furthermore, it is crucial to identify and deploy effective health education strategies, particularly among underserved populations. Valle et al.'s^12^ work illustrates how visual narrative educational tools, such as fotonovelas, have successfully enhanced the understanding of Alzheimer's disease among Spanish‐speaking Latino older adults. However, as Rostamzadeh et al.^13^ points out, the field of Alzheimer's disease is rapidly evolving, with technologies increasingly focusing on early detection and prediction. This constant evolution and influx of intricate information necessitate robust health literacy skills, particularly for individuals at high risk of developing the disease.^13^ Therefore, understanding and interpreting this complex risk information is vital for making informed health decisions. As such, scholars should adopt a systematic approach to address this problem by developing strategies that consider individuals' unique needs and preferences.^13^

Several studies in Japan have analyzed the materials intended to provide health information.^5^ The *Maternal and Child Health Handbook*, a multidimensional health information handbook for long‐term practical use in Japan, is a representative example. This handbook provides health information for parents, children and physicians and is used as a valuable resource from pregnancy until the child is 6 years old and starts school. It is a useful two‐way tool to improve communication between parents and healthcare providers, ensuring continuity of care.^14^ However, the health handbooks regarding dementia have not been as effective. A review of 14 health handbooks published in Japan revealed that all three handbooks that focused on dementia scored below average in health literacy owing to poor clarity and usability.^5^ Therefore, there is an urgent need to develop an effective handbook for dementia prevention among older adults with MCI.

The current study utilized a participatory research approach and conducted formative assessments to develop a health information handbook tailored for older adults with MCI that explains dementia prevention and encourages healthy living. The primary aim of this handbook is to enhance dementia literacy and health literacy among older adults and their families, providing them with the knowledge and tools necessary for informed care and self‐management. This article describes the process of creating this handbook to facilitate the development of effective materials that cater specifically to the health information needs of older adults with MCI.

## 2 METHODS

### 2.1 Participatory approach

The MCI handbook was developed by employing a participatory research approach involving formative assessments to foster the comprehension of dementia‐prevention strategies and promote healthy living among individuals with MCI. The development of this handbook drew on the successes of an approach utilized in 2018–2019 to facilitate evidence‐based dialogue between residents and healthcare professionals regarding radiation after the Fukushima nuclear accident.^15^ Conforming with the principles of participatory research, this study involved patients diagnosed with MCI and their family members to ensure that the research process and outcomes were not only meaningful but also applicable and beneficial to those most affected by MCI. Participatory research, as conceptualized by Cornwall,^16^ is a methodology where the research subjects (in this case, MCI patients and their families) actively participate throughout the research process, including designing the study, formulating research questions, collecting and analyzing data and disseminating results. Emphasizing the importance of this approach, Greenwood et al.^17^ noted that it significantly enhances the validity and relevance of research findings and promotes a sense of ownership and responsiveness among the participants. Our study incorporated participatory research principles in several ways. First, we conducted semi‐structured interviews with five patients with MCI and a family member of each patient. This approach allowed for an accurate reflection of their experiences and information needs. Furthermore, it promoted an environment in which they felt heard and empowered. Second, patients with MCI were given the opportunity to participate in a clinical study utilizing the developed handbook. This step ensured that the handbook genuinely resonated with the lived experiences and information needs of the MCI patients and their families, which underscores the essence of participatory research.

### 2.2 Development process

**Figure 1:**
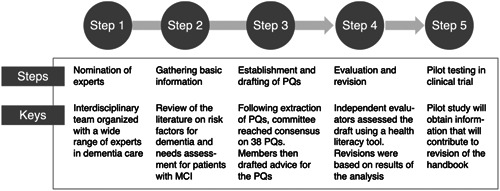
Process of creating a handbook using participatory methods. PQ, patient question.

*Step 1. Nominating the experts*: We assembled an interdisciplinary team of experts, including physicians (H. A. and T. S.), physical therapists (T. S., K. F., K. U., H. S.), occupational therapists (N. M.), social welfare specialists (A. S.), psychologists (Y. K.), designers/editors (I. G. and H. M.) and communication specialists (A. G.) with a wide range of experience in dementia care, covering aspects such as lifestyle, exercise, nutrition, psychological support, cognitive training, social participation and social work (Figure 1).

*Step 2. Gathering basic information*: Considering the dementia risk factors indicated in reports from the World Health Organization (WHO) and the Lancet Commission,^18^ we conducted an extensive review of relevant literature using medical databases, including MEDLINE. An abstract table was created for compiling the findings from the cited literature. Additionally, we interviewed five patients diagnosed with MCI and a family member of each patient to develop a set of patient questions (PQ) regarding care from the patient's perspective.^19^

*Step 3. Establishing and drafting the PQs*: In total, 38 PQs were developed based on the information gathered from the literature review and interviews. Initially, we reviewed relevant sources such as the Lancet Committee's findings on the risk of dementia, the WHO report addressing the same topic, and subsequently, published articles^18^, ^20^ for developing the initial set of PQs. Furthermore, the interviews with patients and their families led to the creation of a dedicated ‘family’ section, incorporating four PQs regarding family‐related concerns. A consensus‐building meeting was held with additional experts to ensure that the PQs were comprehensive. The experts also provided valuable insights and feedback on each PQ and noted that the explanations should be evidence‐based and the language should be easy to understand without using excessive technical jargon and should be accompanied by relevant illustrations to enhance understanding.

*Step 4. Evaluating and revising the handbook*: To assess the prepared advice, five graduate students specializing in community health independently rated the clarity of the materials against the Clear Communication Index (CCI).^21^ Additionally, free‐text responses were obtained from the participants regarding the ‘points of difficulty’ in understanding the material. Based on the analysis results, the team of experts and editors made necessary revisions and information text boxes were created within the reading materials to aid reader understanding.

*Step 5. Pilot testing in a clinical trial*: An intervention test with the handbook will be conducted among individuals with MCI to obtain insights for revising the handbook to best promote behavioural changes. We are currently conducting a 1‐year pilot test in a clinical trial setting, with 30 individuals diagnosed with MCI as participants. The primary outcome measure of this trial is the change in participants' lifestyle behaviours. As a secondary outcome, we are evaluating the quality of the handbook itself, focusing on its usability, comprehensibility and comprehensiveness. In addition, we are investigating various situations where the handbook could be effectively utilized.

### 2.3 Evaluation of the handbook: Formative assessment

1. *Interviews with patients and their family members*: Semi‐structured interviews were conducted with five patients diagnosed with MCI and five corresponding family members. These interviews aimed to gather foundational data for PQ selection and to understand their specific information needs concerning MCI. The interviews included questions such as ‘What information have you received about dementia?’, ‘What resources do you utilize to gather information (such as books, internet, social networking services, etc.)?’, ‘What content about dementia prevention do you find necessary?’, and ‘What issues have you encountered in daily life due to cognitive decline?’. With the interviewees' permission, the responses were transcribed and coded into thematic codes by a researcher experienced in qualitative research.^22^ These coded results were then shared with the development team for further analysis and application.
2. *Evaluation using health literacy tools*: To quantitatively evaluate the draft handbook in promoting health literacy, two tools were used: the CCI and the suitability of assessment materials (SAM).^23^ In addition, to obtain qualitative feedback, the team asked the evaluators to provide free‐text descriptions of the ‘difficult‐to‐understand’ aspects and ‘areas for improvement’ in the materials. The CCI and free‐text descriptions were used to comparatively evaluate the draft and revised versions of the handbook to identify whether the revisions helped improved the handbook's ease of understanding. The evaluators comprised five graduate students majoring in health sciences, who were interested in this topic. The CCI is a tool developed by the United States' Centers for Disease Control and Prevention (CDC) to determine the effectiveness of the information communicated.^21^ The Japanese version, translated by Goto et al.,^24^ consists of 19 items focusing on content, language, design, scientific aspects and behaviour change. Each evaluation item is rated as either 0 (poor) or 1 (good), and the final score (percentage) is calculated as 100% × the total score ÷ the total number of points possible. Higher scores indicate that the material is more easily understood, with a score of ≥90% considered desirable according to the CDC manual. The SAM is a tool used for objectively evaluating the overall comprehensibility of a text.^23^ In this study, we primarily focused on the emotional consideration aspect because the CCI already includes other aspects. Results were scored similarly to the CCI, ranging from 0 to 2, with higher scores indicating better comprehension of the entire text. In the SAM manual, resources are categorized as superior, adequate, or unsuitable based on their scores falling within 70%–100%, 40%–69% and 0%–39%, respectively. Each PQ was evaluated using the CCI and the SAM by the five evaluators. The scores were then averaged and categorized based on the relevant manuals (CCI or SAM), and the frequency and percentage were calculated for each category. The evaluators' procedures were standardized by consulting the guidelines provided in the CCI and SAM evaluation manuals.
  Text mining of the free‐text descriptions was performed using the KH Coder v. 3 software.^25^ First, a list of frequently occurring words and phrases was compiled for both the pre‐ and postrevision versions, and a subgraph analysis of the co‐occurrence network was performed to classify the words and phrases into major topics. The strength of association in the co‐occurrence network was calculated using the Jaccard coefficient. The top 60 strongest associations were drawn on a diagram, and closely related words were colour‐coded.
3. *Opinions of members involved in the development*: After the draft commentary of the guide was prepared, a Web conference was held to facilitate consensus among the developers. During this conference, experts who wrote the commentary draft explained its content and key points, while other experts offered their opinions on its appropriateness and suggested improvements. Communication experts further discussed how experts, patients and families could share and interactively use health information through the handbook.

### 2.4 Ethical considerations

The Institutional Review Board reviewed and approved all study procedures (No. 1603), and the protocol has been registered with the Clinical Trials Registry (UMIN‐CTR) of the University Hospital's Medical Information Network, under registration number UMIN000048338.

## 3 RESULTS

### 3.1 PQ selection and domains

A total of 38 PQs were selected from nine domains (basic information on MCI, lifestyle, exercise, nutrition, social participation, cognitive training, lifestyle‐related diseases, psychological care and family support) (see Supporting Information Table). In addition to the domains proposed by the Lancet Commission report, we introduced two domains: cognitive function training and social participation, which were supported by newly accumulated evidence relevant to people diagnosed with MCI. Additionally, based on the results of the interviews with family members, we included the family members domain, which is a unique domain that addresses specific family concerns.

All the PQs are listed in the Supporting Information Table.

### 3.2 Assessment of understanding: Pre‐ and postrevision comparisons

**Figure 2:**
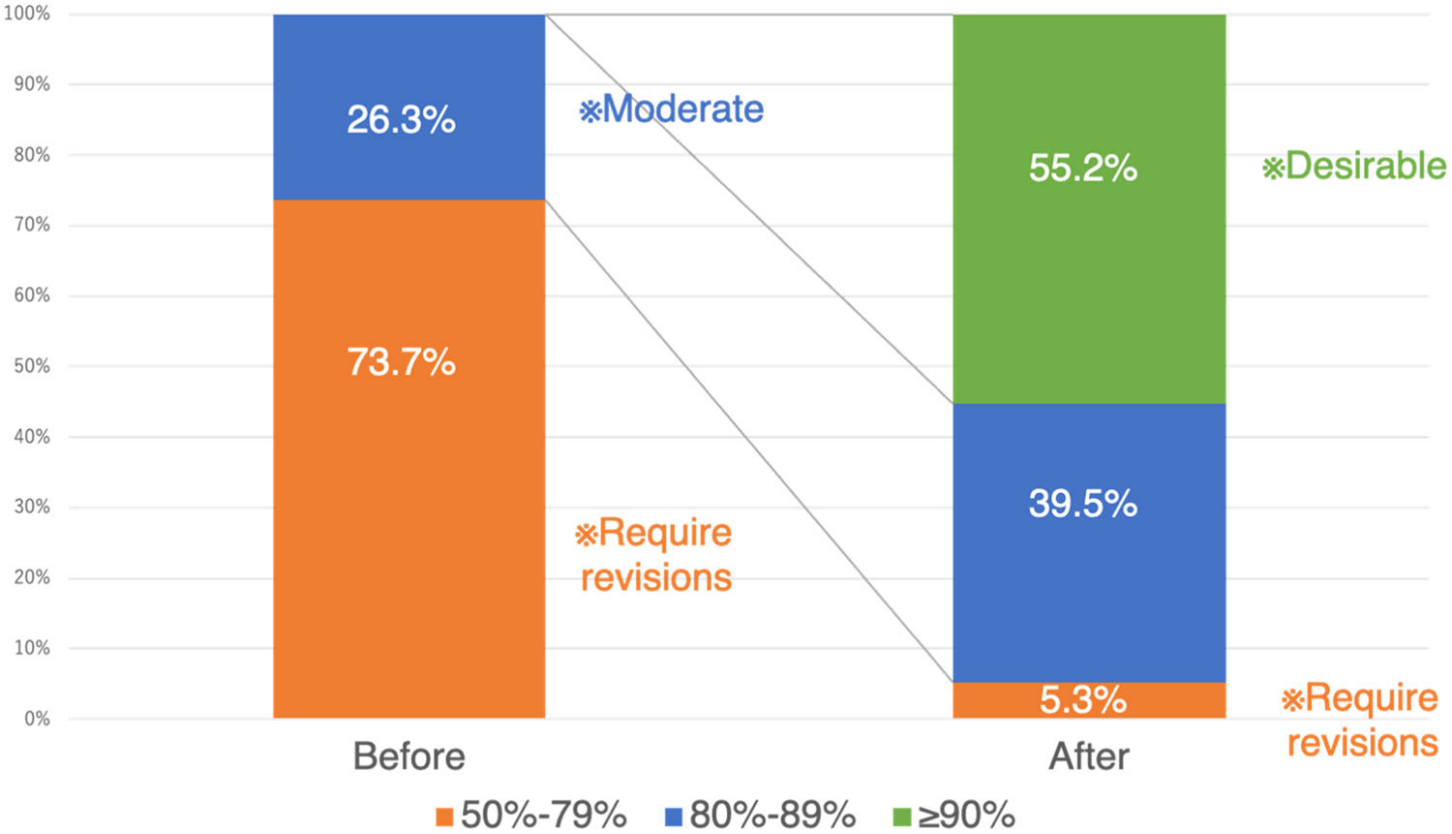
Evaluation of the handbook based on the Clear Communication Index before and after revision.

A comparison of the CCI ratings (Figure 2) showed that before the revision, 73.7% of the PQs were rated as difficult to understand, 26.3% as moderately easy to understand and none were rated as easy to understand, with the scores ranging from 59% to 85%. In contrast, after the revision, only 5.3% of the PQs were rated as difficult, 39.5% as moderately easy and 55.2% as easy to understand, with scores ranging from 79% to 96%. The SAM index was used to rate the emotional considerations aspect after the revision, and all PQs were rated as 70% or above (superior).

### 3.3 Key revision findings: Text mining of points of difficulty

**Figure 3:**
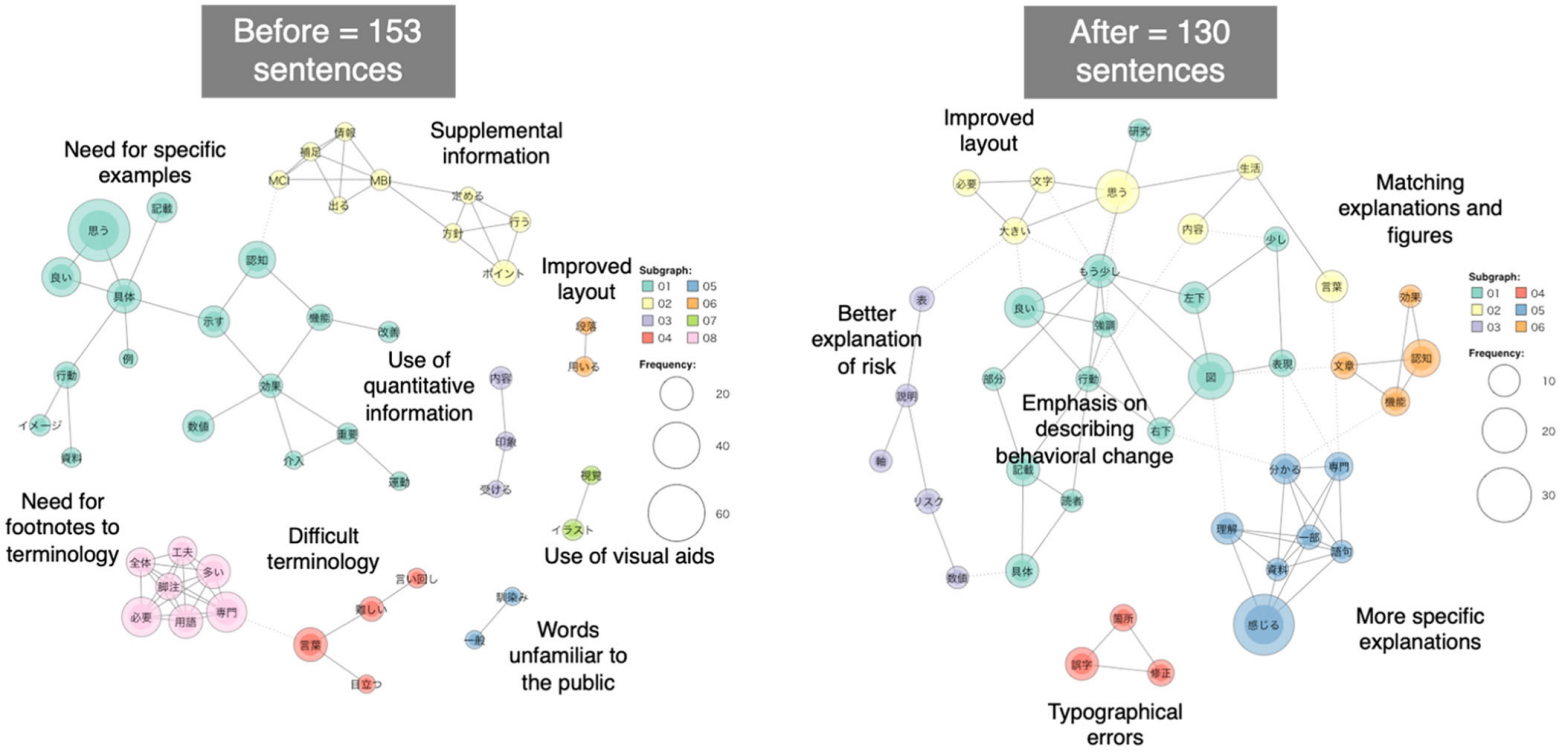
Results of text mining of free descriptions (areas for improvement) before and after revision.

During the text mining, the number of frequently used words and phrases was 153 before the revision and 130 after the revision (Figure 3). The extracted main topics were colour‐coded into eight categories before the revision (improved layout, use of quantitative expressions, use of visual aids, words unfamiliar to the public, difficult terminology, need for footnotes to terminology, need for specific examples and supplemental information) and six after revision (matching explanations and figures, more specific explanations, typographical errors, emphasis on describing behavioural change, better explanation of risk and improved layout). Before the revision, there was a need for more specific explanations, whereas after the revision, the points raised were mostly regarding editing aspects, such as typographical errors and ensuring consistency between figures and explanations.

### 3.4 Strategies implemented to improve reader understanding

Based on the results of the formative assessment, the development team made the following revisions to facilitate reader understanding:

1. *Easy‐to‐understand explanations*: Enlarged text was used for sections likely to be read by older adults, who are often the primary audience. The text was presented in simple language for clarity. In addition, illustrations were used to explain important concepts. An abstract table was included in a separate volume for those desiring further details.
2. *Creation of boxes with first‐person storytelling*: We introduced boxes to present the experiences of people with similar symptoms and circumstances alongside advice from an exercise instructor and a nutritionist on lifestyle behaviour changes.^26^
3. *Development of a lifestyle notebook*: We created a comprehensive checklist that could be easily shared with others (e.g., in group settings) for tracking daily goals, exercise routines, diet and other aspects to encourage healthy lifestyle habits.
4. *Design innovations*: To reduce the readers' cognitive burden, we used three basic colours, eliminated terms such as ‘dementia’ from the cover, as they are often avoided by older adults, adopted a cartoon‐style presentation to attract interest, and introduced a character who serves as a guide throughout the book.

## 4 DISCUSSION

### 4.1 Evaluation of the dementia prevention handbook

To the best of our knowledge, this was the first handbook on dementia prevention that specifically targeted individuals diagnosed with MCI and was developed using a participatory research approach. We used the CCI to assess the comprehensibility and readability of the handbook, which improved significantly after revision. In a previous study evaluating handbooks on child care, the CCI scores ranged from 40% to 80% with a median of 67%.^24^ In another study evaluating 14 health handbooks published in Japan, the CCI scores ranged from 69% to 100% with a median score of 81.5%.^5^ The revised CCI scores for the PQs in the current handbook ranged from 79% to 96%, suggesting that our handbook scores favourably among health handbooks. However, as the recommended CCI score is ≥90%, there is still scope for further improvement. Given that health information serves a similar function as drug therapy and medical intervention, the handbook should be carefully evaluated and revised before its release.^27^

### 4.2 Participatory approach in developing the handbook

In the development of our practical handbook on dementia prevention, a participatory approach involving patients and their families was implemented, following the recommendations of a communication specialist in our team.^28^, ^29^ This method allowed for meaningful two‐way communication between the experts involved in the study and the actual readers of the handbook. Thus, it facilitated a dynamic dialogue to ensure that the revisions authentically reflected the voices of the potential readers of the handbook.^30^ One prominent advantage of this participatory approach was the opportunity to engage in discussions and Q&A sessions, revealing that the concerned parties were keenly interested in learning from the experiences of people with similar symptoms and circumstances. This exchange not only enhanced their knowledge but also provided valuable insights into shared experiences, such as visiting a hospital regarding concerns about forgetfulness, adopting exercise, monitoring diet and exercise to improve lifestyle, and experiences of older persons living alone in communicating with distant family members using social networking services. Recognizing the importance of these shared experiences, our team of experts incorporated these real‐life examples into the handbook, including practical tips and actionable insights. This not only enriched the content of the handbook but also fostered a sense of community and empathy among readers, bridging the gap between clinical understanding and the lived experiences of people with the condition.^31^

Furthermore, adopting a participatory approach allowed us to create a document that was both scientifically grounded and personally resonant.^32^ The inclusion of firsthand accounts contributed to a more nuanced understanding of the disease's progression and the practical challenges it poses. In addition, it emphasized the social and human aspects of dementia care, strengthening the handbook's relevance and applicability for potential users. However, this approach was not without its challenges, particularly the time‐consuming nature of engaging with patients with dementia.^33^, ^34^, ^35^ Nonetheless, the tangible benefits of this participatory research, including the alignment with real‐world perspectives and the incorporation of practical advice, have substantially contributed both to the scholarly field and the lives of those affected by dementia.^33^ In conclusion, the participatory approach employed for developing this handbook represents a powerful synergy between research rigour and compassionate understanding. It has not only advanced our scientific perspectives on dementia but has also crafted a living document, informed by the very people it aims to serve, enriched with practical wisdom and communal empathy.

### 4.3 Health literacy addressed in the handbook

Our study has noteworthy implications for health literacy research on dementia. While existing literature has shown that low health literacy is associated with poorer health outcomes and higher healthcare costs,^36^, ^37^ the focus on health literacy in dementia care has been relatively limited. This is a concerning issue, especially considering the complexities and challenges faced by people with dementia and their caregivers in understanding, managing and making informed health decisions.^38^ To address this research gap, experts in gerontology, longevity science and health literacy collaborated in developing this handbook, utilizing the CCI to integrate health literacy principles. This collaboration facilitated substantial revisions aimed at enhancing the content's readability, accessibility and relevance for individuals with MCI. The key changes included: (1) emphasizing visual content and actions for the target audience; (2) using consistent and familiar language; (3) structuring the layout with headings, bullet points and summarizing key information; (4) clearly explaining both the source information and current scientific understanding; (5) presenting numerical information in familiar terms; and 6) adopting a ‘one‐size‐fits‐all’ format. Additionally, the handbook incorporated a lifestyle notebook for daily lifestyle monitoring and goal setting, including free comments. This feature facilitates two‐way communication between individuals with MCI and their supporters, thus fostering a practical application of health literacy concepts. Collectively, these refinements transformed the handbook into a resource prioritizing the needs of its readers, aligning with best practices in health literacy, and serving as a practical guide for those affected by MCI and their caregivers.

Our study underscores the importance of integrating health literacy principles into dementia research and care. By ensuring that the dementia prevention handbook is understandable, accessible and relevant to people with MCI, we have taken a crucial step toward improving their health literacy. The participatory approach employed positions individuals with MCI as active participants in their health care, rather than treating them as passive recipients of care. In addition, promoting health literacy in dementia care has broader implications for the healthcare system, including policy, practice and education. Healthcare providers and educators need to be equipped with effective communication skills to interact with people with dementia and their caregivers. Policymakers should prioritize health literacy and promote initiatives and resources supporting the enhancement of health literacy among patients with dementia and their families.

### 4.4 Limitations of the study

Although the present study reveals important findings, it has several limitations. One limitation is that while participatory approaches are commonly used in multiple stages of the research, such as research design and planning, data collection and analysis, publication and practical application of results, this approach was utilized only in some stages of the study. Except for the study by Parveen et al.,^39^ participant involvement in much of the successful participatory dementia research has been partial, mostly covering the data collection and analysis stages. While highlighting the costly and time‐consuming challenges of working with people with dementia, Parveen et al.^39^ conclude that the relationship between patients and researchers is key to achieving success. Currently, we are conducting a participatory pilot study involving 33 patients with MCI (UMIN000048338) to further examine the practical usage and effectiveness of the developed handbook. Another limitation is that health literacy has not been directly assessed by people with MCI. While previous studies have shown that self‐reports from people with MCI may not be entirely accurate, others have suggested that they can be used in domains other than those measuring cognitive function.^40^ As the CCI is designed to be used by a wide range of people, future evaluations by people with MCI could provide a more comprehensive understanding of the actual situation.^21^ In addition, the present study employs both quantitative and qualitative methodologies, which provides valuable insights. However, its limitation lies in not leveraging a mixed‐method approach, which could amalgamate the strengths of each method. Finally, the results of this study indicate that some PQs did not meet the criteria for a good score on the CCI. Therefore, in the future, we plan to have people with MCI evaluate the handbook using tools such as CCI and make necessary revisions based on their feedback.

### 4.5 Conclusion and recommendations for future research

In conclusion, this study underscores the significant progress made in developing the first dementia‐prevention handbook tailored for individuals with MCI, particularly highlighting the value of the participatory research approach. Despite certain limitations, efforts to improve the CCI score continue, emphasizing the importance of making health information accessible and relevant to those affected. Our research illuminates the critical need for including patients' perspectives and advancing health literacy in dementia research.

Future studies should consider adopting a mixed‐method approach and the active involvement of individuals with MCI to bolster the comprehensibility and efficacy of health interventions. As we navigate the complexities of dementia prevention, our primary goal remains to enhance the quality of life for individuals with dementia and their caregivers. Our project serves as a model to provide a mechanism for patients with MCI and their families to identify and express their needs, allowing professionals to support them more efficiently, and promoting effective collaboration among all stakeholders.

## AUTHOR CONTRIBUTIONS

Yujiro Kuroda and Takashi Sakurai designed the study and planned recruitment. Yujiro Kuroda and Aya Goto performed quantitative and qualitative analyses and wrote first draft. Taiki. Sugimoto, Kosuke Fujita, Kazuaki Uchida, Nanae Matsumoto, Hiroyuki Shimada, Rei Ohtsuka, Minoru Yamada, Yoshinori Fujiwara, Aya Seike, Hidenori Arai drafted the handbooks and Gabin Ito and Madoka Hattori revisited them. All authors contributed to the interpretation and discussion of the results and approved the submitted manuscript.

## CONFLICT OF INTEREST STATEMENT

The authors declare no conflict of interest.

## Supporting information

Supporting information.

## Data Availability

The data that support the findings of this study are available on request from the corresponding author. The data are not publicly available due to privacy or ethical restrictions.
