# Supplementary material for: Participatory approaches for developing a practical handbook integrating health information for supporting individuals with mild cognitive impairment and their families
Source: Health Expect. 2023 Sep 19;27(1):e13870. doi: 10.1111/hex.13870 (PMC10726060; doi:10.1111/hex.13870)
Supplement: Supplementary file 1 — Supporting information. [file HEX-27-e13870-s001.docx]

**Supplementary Table.** Patient questions by dimension.

| **Basic information on mild cognitive impairment (MCI)** |
| --- |
| Q1. What are the symptoms of dementia? How does it differ from age-related forgetfulness?  Q2. What are the causes of dementia? What treatment options are available?  Q3. What is MCI? |
| **Lifestyle-related diseases** |
| Q4. I was told that I have diabetes. Am I more likely to develop dementia?  Q5. I have been visiting the hospital for hypertension. What is its relationship with dementia?  Q6. Can being overweight or underweight lead to dementia?  Q7. I am visiting the hospital owing to a stroke. Could I be at risk of developing develop dementia?  Q8. I was told that my lipids (cholesterol and neutral fat in my blood) are higher than the standard. How is this is related to dementia? |
| **Exercise** |
| Q9. Can regular exercise reduce the risk of developing dementia?  Q10. Does exercise improve cognitive function?  Q11. Does the effect on cognitive function differ depending on the type of exercise?  Q12. How much and what kind of exercise should I do per week to improve my cognitive function? |
| **Nutrition** |
| Q13. Can the progression of MCI be inhibited by diet?  Q14. What kind of diet can prevent MCI progression?  Q15. Can I inhibit the progression of MCI by taking nutritional supplements?  Q16. Are there any nutritional considerations for individuals with MCI and dementia and their families? |
| **Social participation** |
| Q17. I have been living without a daily routine since I quit my job, and I am worried that I may develop dementia. What activities do you recommend?  Q18. I heard that it is important to talk with people. What is the relationship between interaction with others and dementia?  Q19. Is it better to go out than to stay at home all day? How much should I go out?  Q20. I am worried because I can’t think of anyone who can help me when I am in trouble. What should I do? |
| **Cognitive function training** |
| Q21. Is playing brain-training games regularly effective for improving the cognitive function of older adults with MCI?  Q22. Is cognitive training at a specialized institution effective in preventing cognitive decline?  Q23. Are board games such as mahjong and go effective in improving cognitive function?  Q24. Do musical activities help improve cognitive function?  Q25. Do artistic activities (cultural experiences such as creative writing and appreciation) help improve cognitive function? |
| **Lifestyle** |
| Q26. What is the relationship between smoking and dementia?  Q27. I heard that drinking too much alcohol makes one more susceptible to dementia. What is the relationship between alcohol and dementia?  Q28. Is it easier to get dementia if I am hard of hearing?  Q29. I have trouble falling asleep and waking up in the middle of the night. Does it make me more prone to dementia? |
| **Psychological care** |
| Q30. Lately, I have been perplexed by sudden depression, irritability, and extreme ups and downs in my mood. What should I do?  Q31. I have been diagnosed with MCI, but I feel as though I am the only one who has not been informed of anything, and I am worried. Can you provide more details about the treatment methods and expected course of treatment?  Q32. When I tell my family about my upbringing and my experiences when I was working, they say to me, “I’ve heard this so many times already! Don’t tell me anymore.” Should I stop telling my family about my past?  Q33. Can I take care of myself or should I apply for long-term care insurance? |
| **Family support** |
| Q34. Recently, I have become angry, apathetic, and sometimes pessimistic and depressed, as though I am a different person. What is causing this?.  Q35. Recently, I am worried that the person has become forgetful and suspect that he/she may have dementia. When we visit medical institutions, should we see them separately and not tell the person about the diagnosis?  Q36. My partner, who has been diagnosed with MCI, has trouble controlling his/her feelings and is verbally abusive. How do I deal with him/her better?  Q37. Since being diagnosed with MCI, the patient has been telling me the same story over and over every day, and it is bringing me down. I want to listen to him carefully, but I feel frustrated. Is there any way to help both the patients and their families?  Q38. The person is able to take care of him/herself, but I was advised to apply for long-term care insurance. What type of insurance should I apply for? |
| **Boxes** |
| Let’s learn about the relationship between lifestyle-related diseases and dementia!  Don’t be misled! Commonly heard misconceptions about dementia  How do I find the exercise that is right for me?  How can I create a cycle of enjoyable eating?  Items to help prevent forgetting things  Try brain-training games!  What day is it today? What are your plans? Technology that informs us  How to avoid trouble shopping.  Experiences of people with similar symptoms and circumstances alongside advice from an exercise instructor and nutritionist on how to change behavior. |
